# Dehydroglyasperin D Suppresses Melanin Synthesis through MITF Degradation in Melanocytes

**DOI:** 10.4014/jmb.2207.07043

**Published:** 2022-08-01

**Authors:** Eun Ji Baek, Yu-Bin Ha, Ji Hye Kim, Ki Won Lee, Soon Sung Lim, Nam Joo Kang

**Affiliations:** 1 School of Food Science and Biotechnology, Kyungpook National University, Daegu 41566, Republic of Korea; 2 Department of Agricultural Biotechnology, Seoul National University, Seoul 08826, Republic of Korea; 3 Department of Food Science and Nutrition, Hallym University, Chuncheon 24252, Republic of Korea

**Keywords:** Licorice, dehydroglyasperin D, melanin, microphthalmia-associated transcription factor, melanocytes

## Abstract

Licorice (*Glycyrrhiza*) has been used as preventive and therapeutic material for hyperpigmentation disorders. Previously, we isolated noble compounds including dehydroglyasperin C (DGC), dehydroglyasperin D (DGD) and isoangustone A (IAA) from licorice hexane/ethanol extracts. However, their anti-melanogenic effects and underlying molecular mechanisms are unknown. The present study compared effects of DGC, DGD and IAA on pigmentation in melan-a melanocytes and human epidermal melanocytes (HEMn). DGD exerted the most excellent anti-melanogenic effect, followed by DGC and IAA at non-cytotoxic concentrations. In addition, DGD significantly inhibited tyrosinase activity in vitro cell-free system and cell system. Western blot result showed that DGD decreased expression of microphthalmia-associated transcription factor (MITF), tyrosinase and tyrosinase-related protein-1 (TRP-1) in melan-a cells and HEMn cells. DGD induced phosphorylation of MITF, ERK and Akt signal pathway promoting MITF degradation system. However, DGD did not influence p38 and cAMP-dependent protein kinase (PKA)/CREB signal pathway in melan-a cells. These result indicated that DGD inhibited melanogenesis not only direct regulation of tyrosinase but also modulating intracellular signaling related with MITF level. Collectively, these results suggested a protective role for DGD against melanogenesis.

## Introduction

Melanin is a dark-brown pigment that determines the color of skin, hair and eyes. In the mammalian epidermis, melanocytes synthesize the melanin to protect the skin against ultraviolet (UV)-induced DNA damage [1]. However, excessive accumulation of melanin in the skin can cause hyperpigmentary disorders such as melasma, freckles and age spots [2, 3].

Tyrosinase, tyrosinase-related protein-1 (TRP-1) and tyrosinase-related proteins-2 (TRP-2) are key enzymes involved in melanin synthesis in mammalian melanocyte [4]. Tyrosinase catalyzes the oxidation of L-tyrosine to 3,4-dihydroxyphenylalanine (L-DOPA), and L-DOPA is converted to dopaquinone is the first step in melanin synthesis. After then, dopaquinone is oxidated by TRP-1 or TRP-2 to finally form melanin. Tyrosinase is the rate limiting enzyme in the process of melanin synthesis, also called melanogenesis. For this reason, inhibition of tyrosinase is considered as effective skin whitening strategy [5].

Melanogenic enzyme expression can be stimulated by various factors such as UV radiation, a-melanocyte stimulating hormone (MSH) and inflammatory cytokines [6, 7]. This event is mainly mediated by microphthalmia-associated transcription factor (MITF) [8]. MITF specifically interact M-box and E-box motif to transactivate the tyrosinase, TRP-1 and TRP-2 gene promoters [8]. MITF is stimulated by cAMP response element-binding protein (CREB) which is induced via the cAMP-dependent protein kinase (PKA) and p38 MAPK signaling pathways [8].

In contrast to PKA and p38 signaling, extracellular signal regulated kinases (ERK) signal pathway negatively regulates MITF level. Activation of ERK1/2 directly phosphorylates MITF on Ser73, which promotes MITF degradation, leading to downregulated tyrosinase expression and suppressed melanin production [8, 9]. Phosphatidylinositol3-kinase (PI3K)/Akt signal pathway is also associated with downregulation of melanin synthesis via phosphorylation of GSK3β, which involved in MITF phosphorylation on Ser209 and inhibit transcription of MITF, leading to inhibition of melanin synthesis [10-12].

**Fig. 1:**
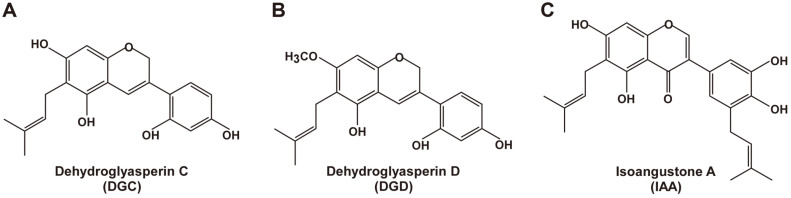
The chemical structure of (**A**) DGC, (**B**) DGD and (**C**) IAA.

Licorice (*Glycyrrhiza*) is a well-known herbal medicine and natural sweetener in candies, chewing gums [13, 14]. Licorice extracts have been used as topical skin whitening agents for the improvement of skin pigmentation disorders [15]. Few compounds derived from licorice extract are reported to suppress melanin synthesis in B16F10 cells via inhibition of tyrosinase activity and expression [16-18]. However, licorice extracts have been reported to possess a large number of components including saponins, flavonoids, isoflavonoids, and chalcones and their biological activities have not been fully understood. Previously, new active flavonoids including dehydroglyasperin C (DGC), dehydroglyasperin D (DGD) and isoangustone A (IAA) (Fig. 1) were isolated from licorice hexane/ethanol extracts [19]. DGC, DGD and IAA exerted numerous biological activities such as anti-oxidation, anti-inflammation and anti-cancer [20-23]. However, there are no report about skin whitening effects of DGC, DGD and IAA.

In the present study, we compared the anti-melanogenic effects of DGC, DGD and IAA in melan-a murine melanocytes. We found that DGD revealed the most outstanding inhibitory effect on melanin production and tyrosinase activity compared to DGC and IAA. Therefore, we focused on the anti-melanogenic effects of DGD using murine melan-a melanocytes, human epidermal melanocytes (HEMn) and zebrafish model.

## Material and Methods

### Materials

DGC, DGD, and IAA were provided by Hallym University in Chuncheon, Korea. RPMI 1640, Medium 254, penicillin-streptomycin and 0.5% trypsin-EDTA were obtained from GIBCO Invitrogen (New Zealand). Human melanocyte growth supplement (HMGS) purchased from Cascade Biologics (USA). 3-(4,5-dimethylthiazol-2-yl)-5-(3-carboxy-methoxyphenyl)-2-(4-sulfonyl)-2H-tetrazolium Htetrazolium (MTT), Fetal bovine serum (FBS), mushroom tyrosinase, L-tyrosine, 3,4-dihydroxy-L-phenylalanine (L-DOPA), sea salts, methyl cellulose, n-phenylthiourea, tricainemethanesulfonate, α-MSH, arbutin, dimethyl sulfoxide (DMSO) and β-actin antibody were purchased from Sigma Chemical Co. (USA). The antibodies against tyrosinase, TRP-1, TRP-2, MITF, phosphorylated ERK1/2 (Glu-4), phosphorylated PKA α/β/γ (Thr-198), total ERK, total PKA, goat anti-mouse IgG-HRP and goat anti-rabbit IgG HRP-conjugated secondary antibodies were purchased from Santa Cruz Biotechnology (USA). Antibodies against phosphorylated p90RSK (Ser380), phosphorylated MEK1/2 (Ser-217/ 221), phosphorylated PI3K (p85 (Tyr458)/p55 (Tyr199), phosphorylated Akt (Ser-473), phosphorylated GSK-3β (Ser-9), phosphorylated CREB (Ser-133), total RSK1/2/3, total MEK1/2, total PI3K, total Akt, total GSK-3β, total CREB and total p38 were purchased from Cell Signaling Technology (USA). Phosphorylated MITF (Ser180/73) was purchased from Aviva Systems Biology Co. (USA). Antibodies against phosphorylated p38 (Tyr-180/Tyr-182) were purchased from BD Biosciences (USA). 12-O-tetradecanoylphorbol-13-acetate (TPA) was purchased from Tocris Bioscience (USA). Skim milk was purchased from MB cell (USA). Fontana-Masson staining kit were purchased from American Master*Tech Scientific, Inc. (USA). The protein assay kit was obtained from Bio-Rad Laboratories Inc. (USA).

### Cell Culture

Melan-a cells, an immortal line of pigmented melanocytes, were kindly provided by Prof. Dorothy C. Bennett (St. George’s Hospital Medical School, UK). Melan-a cells were cultured in RPMI 1640 supplemented with 10%unheated FBS, 1% penicillin/streptomycin, and 200 nM TPA at 37°C in a humidified atmosphere with 10% CO_2_. Melan-a cells were cultured every 3 day and maximal passage numbers of 45 were used for the experiments.

Human epidermal melanocytes derived from moderately pigmented neonatal foreskins (HEMn) were purchased from Cascade Biologics (USA). HEMn cells were cultured in Medium 254 supplemented with HMGS and 1% penicillin/streptomycin at 37°C in a humidified atmosphere with 5% CO_2_. HEMn cells were cultured every 3 day and passage numbers between 4 and 10 were used for the experiments.

### Cell Viability Assay

To estimate the possible cytotoxicity of GF1 tested in this study, the cell viability was determined by MTT assay. Melan-a cells or HEMn cells were seeded 1 × 10^4^ cells/well in 96-well plates with culture media at 37°C in a 5% CO_2_ incubator. After culturing for indicated time (1, 2, or 3 days), 20 μl of MTT solution was added to each well. The cells were then incubated for 2 h at 37°C in the CO_2_ incubator, and the absorbance of the cell culture was measured at 570 nm.

### Measurement of Melanin Content

Melan-a cells (2.5 × 10^4^ cells/ml) were seeded into 6 well plate and incubated for 24 h. After that, each samples were treated with indicated concentration for 3 days. The cells were counted and then disrupted in 1 N NaOH with heating at 80°C for 1 h. The dissolved melanin contents were determined by measuring the absorbance at 405 nm using a microplate reader.

### Mushroom Tyrosinase Activity

In vitro tyrosinase inhibitory activity assay was determined using the method described previously with slight modifications [16, 17]. In briefly, each 5 μl of purified mushroom tyrosinase (Sigma, 2 × 10^3^ units/ml) and 5 μl of the test samples (0, 1, 5 μM) was adjusted with 0.1 M potassium phosphate buffer (PH 6.8) for total volume of 100 μl. After mixing by gentle shaking at 25°C for 10 min, 50 μl of L-tyrosine solution (0.3 mg/ml) was added to a 96 well plate. The plate was incubated at 25 °C for a few min, and then the absorbance was measured at 475 nm using a microplate reader (Sunrise-Basic Tecan, Tecan Austria GmbH 5082 Groding, Austria). The tyrosinase activity was calculated as a percentage to untreated control.

### Intracellular Tyrosinase Activity

Intracellular tyrosinase inhibitory activity was determined using the method described previously with slight modifications [24]. Melan-a cells (2.5 × 10^4^ cells/ml in 60 mm dish) were lysed with PBS containing 1% Triton X-100 after washing twice with ice-cold PBS. Cell lysates were clarified by centrifugation for 5 min at 10,000 g. Each cell lysate for 15 μg protein was mixed with test samples (0, 1, 5 μM), and transferred to a 96 well plate with addition of L-DOPA (15 mM). The plate was incubated at 37 °C for 1 h, and then the absorbance was measured at 475 nm using a microplate reader. The tyrosinase activity was calculated as a percentage to untreated control.

### Western Blotting

Melan-a cells (1.5 × 10^4^ cells/ml) were cultured in a 60 mm dish for 24 h. And then, DGD was treated with or without indicated concentration and time. The cell were lysed in cold lysis buffer (20 mM Tris-HCL (PH 7.5), 150 mM NaCl, 1 mM Na_2_EDTA, 1 mM EGTA, 1% Triton X-100, 2.5 mM sodium pyrophosphate, 1 mM glycerophosphate, 1 μg/ml leupeptin, 1 mM PMSF and a protease inhibitor). The protein concentration was determined using a dye-binding protein assay kit (Bio-Rad Laboratories Inc.), as described in the manufacturer’s manual. A 20-40 μg lysate protein was separated via 10% sodium dodecyl sulfate-polyacrylamide gel electrophoresis (SDS-PAGE) and electrophoretically transferred to a polyvinylidene fluoride membrane (Millipore Crop., USA). After blotting, the membranes were blocked with 5% non-fat skim milk in a Tris buffered saline-T buffer at 4°C overnight and incubated for 2 h with the specific primary antibodies (1:1000). After hybridization with secondary antibodies (1:5000, Santa Cruz Biotech, USA), the protein bands were visualized using an ECL plus Western blotting detection system (Amersham™, USA).

### Fontana-Masson Staining

HEMn cells were treated with indicated samples before being stimulated with α-MSH (100 nM) for 3 days. Intracellular melanin accumulation was visualized by Fontana-Masson staining according to the manufacturer’s instructions [25]. Cell morphology and pigmentation were examined under a Nikon phase-contrast microscope (Japan). The images were analyzed using NIS-Elements 3.0 software.

### Statistical Analysis

Where applicable, the data are expressed as means ± SD; Student’s *t*-test was used for single statistical comparisons. A probability value of *p* < 0.05 was used as the criterion for statistical significance. Significant differences were determined by one-way analysis of variance (ANOVA) followed by Dunnett’s post hoc test for comparisons among more than three groups using GraphPad Prism version 8 software (GraphPad Software, USA). Values of *p* < 0.05 were regarded as significant.

## Results

### Cytotoxic Effects of DGC, DGD and IAA on Melan-a Cells and HEMn Cells

**Fig. 2:**
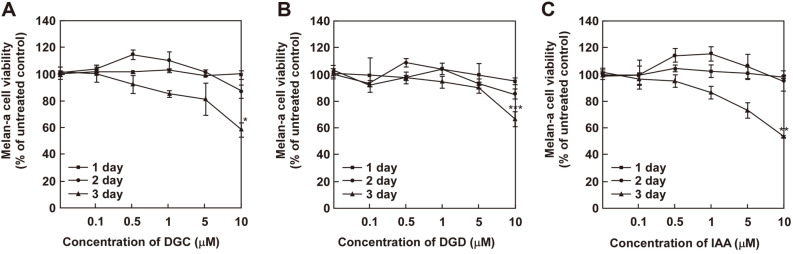
Effect of (**A**) DGC, (**B**) DGD and (**C**) IAA on cell viability in melan-a melanocytes. Cells were treated with each samples at the indicated concentrations (0, 0.5, 1, 5, and 10 μM) and times (1, 2, and 3 day). Cell viability was then determined to MTT analysis as described in the materials and methods.

**Fig. 3:**
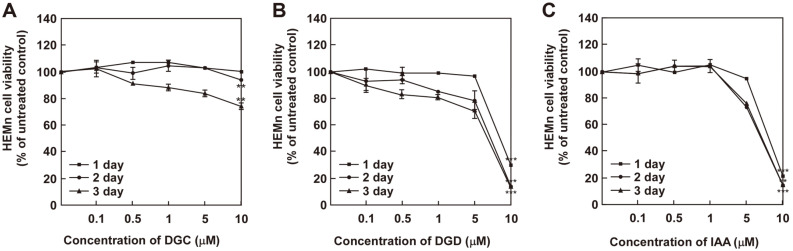
Effect of (**A**) DGC, (**B**) DGD and (**C**) IAA on cell viability in human epidermal melanocytes (HEMn cells). Cells were treated with each samples at the indicated concentrations (0, 0.5, 1, 5, and 10 μM) and times. Cell viability was then determined to MTT analysis as described in the materials and methods. The asterisk (*) indicates a significant difference (**p < 0.05, **p < 0.01) compared with untreated control.

We first determined that the cytotoxic effects of DGC, DGD and IAA in melan-a cells and HEMn cells using MTT assay (Figs. 2 and 3). DGC, DGD and IAA did not significantly affect cell growth at 1, 2, or 3 days after treatment at concentrations up to 1 μM (Figs. 1 and 2).

### Effects of DGC, DGD and IAA on Melanogenesis

**Fig. 4:**
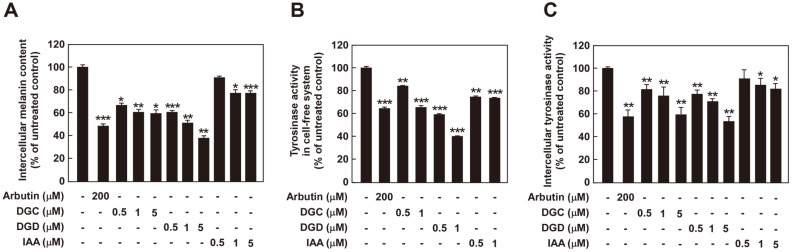
Effect of DGC, DGD and IAA on melanogenesis. (**A**) Effects of DGC, DGD and IAA on melanin production in melan-a cells. Cells were treated with samples at the indicated concentration (0, 0.5, 1, 5 μM) for 3 days. Results are expressed as melanin content to untreated control. (B, C) Effects of DGD on mushroom tyrosinase activity and intrarcellular tyrosinase activity. Results are expressed as tyrosinase activity to untreated control. Data are presented as the mean ± SD of three independent determinations. *p < 0.05, **p < 0.01, ***p < 0.001, one-way repeated measures ANOVA with Dunnett’s multiple comparisons test.

At non-cytotoxic concentration, melanin content assay showed that DGC and DGD treatment significantly decreased melanin level in a dose-dependent manner (Fig. 4A). Next, we examined the inhibitory effect of tyrosinase activity using mushroom tyrosinase and intercellular tyrosinase activity assay. DGD showed the most significantly inhibitory effect on in vitro and intracellular tyrosinase activity, followed by DGC and IAA (Figs. 4B and 4C). These results suggest that DGD suppress melanin production through direct regulation of tyrosinase in melan-a cell.

### Effects of DGD on Melanogenic Enzymes and Transcription Factor Expression in Melan-a Cells

**Fig. 5:**
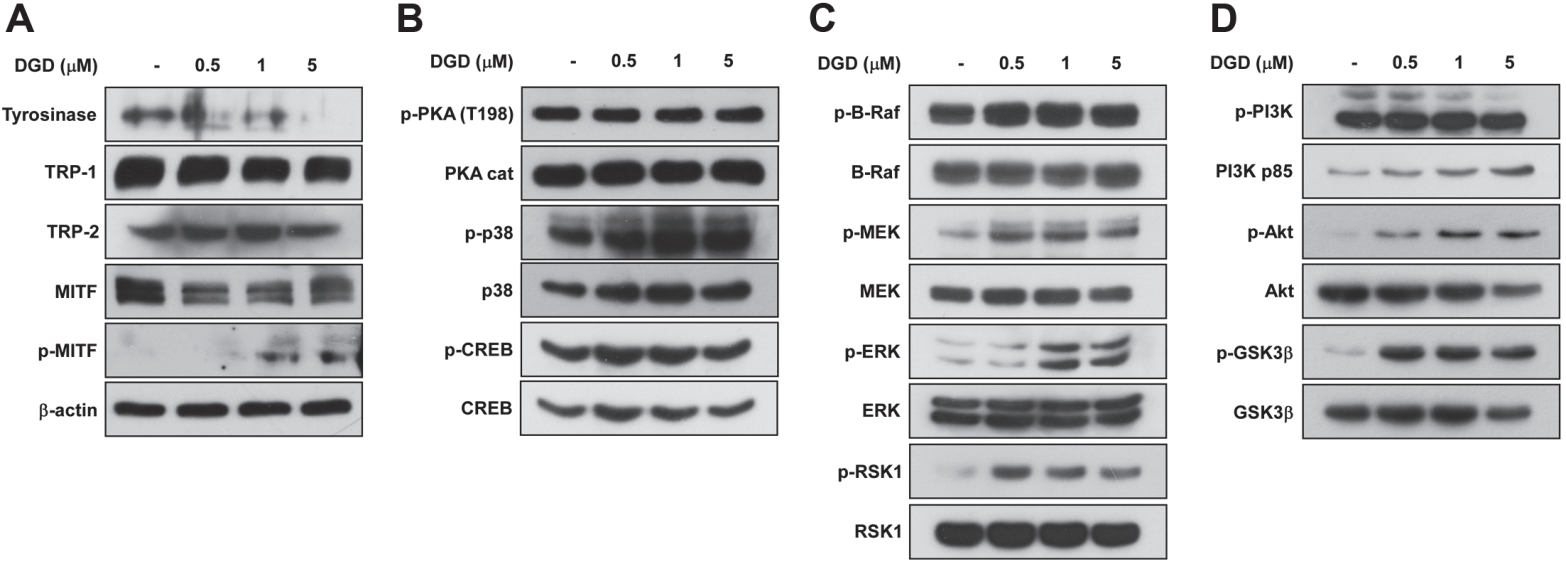
Effects of DGD on melanogenic protein expression and signaling pathway in melan-a cells. (**A**) Melana cells were treated with DGD at the indicated concentrations (0, 0.5 1, 5 μM) for 3 day. Equal protein loading was confirmed using β-actin. Effect of DGD on (**B**) PKA/CREB, (**C**) ERK and (**D**) Akt signaling in melan-a cells. Cells were starved for in serum-free RPMI1640 and treated with DGD at the indicated concentrations (0, 0.5, 1, 5 μM) for 30 min. Cell lysates were then determined to western blot. The data are representative of three independent experiments that gave similar results.

The tyrosinase, TRP-1 and TRP-2 enzymes have known to play important role in melanogenesis. Thus, we determined the whether DGD can inhibit three specific enzyme expression in melan-a cells. DGD significantly reduced tyrosinase and TRP-1 expression at 1 and 5 μM, but not influence TRP-2 expression (Fig. 5A). Next, MITF is transcriptional factor that related with melanin synthesis by activating melanogenic enzyme gene expression. DGD treatment suppressed MITF protein levels and induced phosphorylated MITF levels in a dose-dependent manner. Consequently, these results suggest that DGD reduced melanogenic protein levels through downregulating MITF levels.

### Effects of DGD on Melanogenic Signal Pathways in Melan-a Cells

Previously, p38 and PKA leads to activation of CREB transcriptional factor and consequently enhance MITF gene expression and other melanogenic protein levels. Therefore, we investigated whether DGD reduce phosphorylation of p38 or PKA/CREB in melan-a cells by western blot. As shown in Fig. 5B, DGD did not reduce phosphorylation of p38 and PKA/CREB.

Therefore, we next examined the effects of DGD on other signal pathway including ERK and Akt in melan-a cells. The specific MITF degradation signals derived from ERK activation targeting of Ser73 and Ser409. In addition, MITF degradation was related to Akt signal pathway targeting MITF Ser298. As shown in Figs. 5C and 5D, DGD induced phosphorylation of ERK and Akt signaling in a dose-dependent manner. Overall results indicated that DGD downregulates MITF level by activating ERK and Akt signal in melan-a cells.

### Effects of DGC, DGD and IAA on Melanogenesis in α-MSH-induced HEMn Cells

**Fig. 6:**
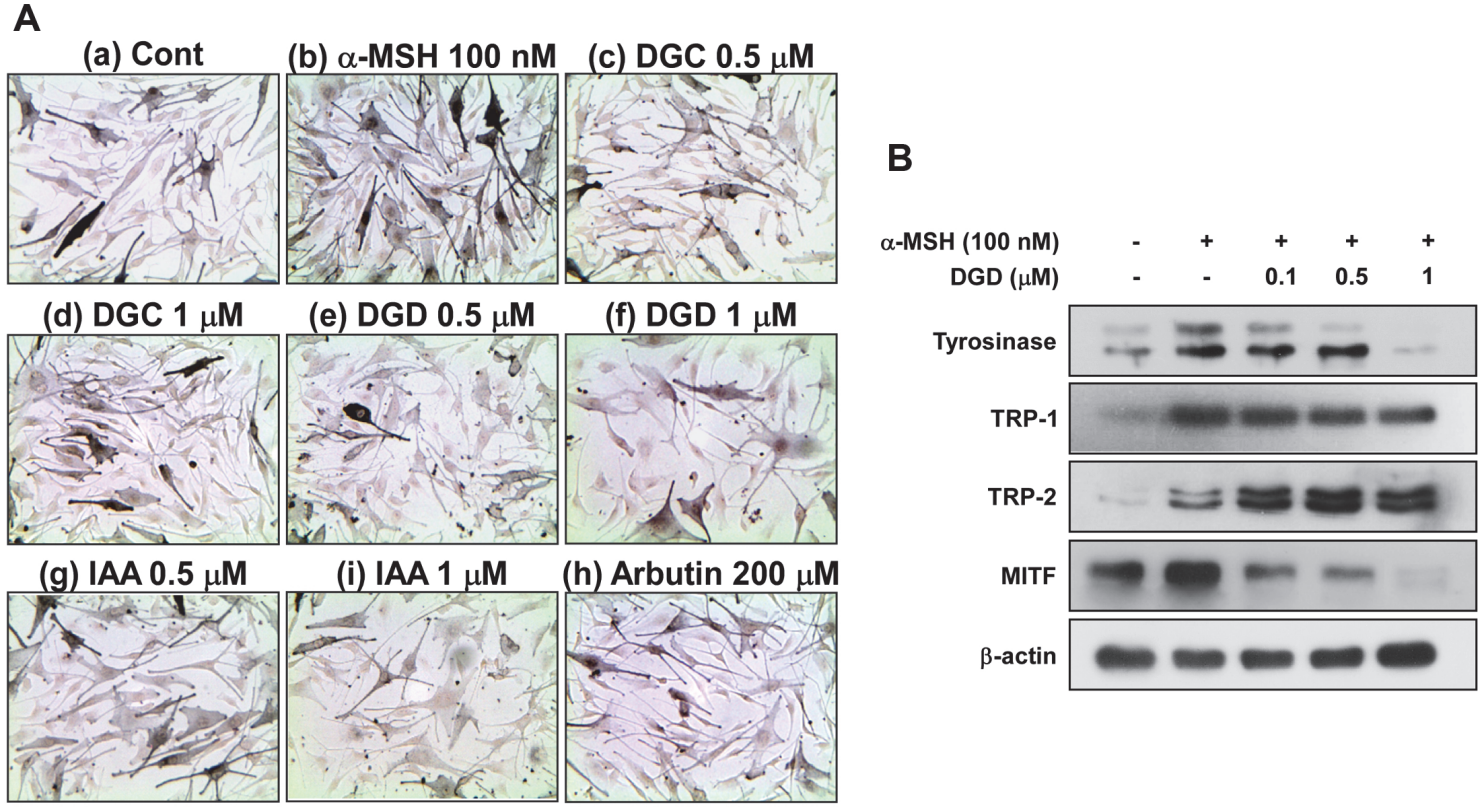
(**A**) Effects of DGC, DGD and IAA on α-MSH-induced melanin content in HEMn cells. HEMn cells were treated with DGC, DGD and IAA for 1 h before being exposed to 100 nM α-MSH. 3 days later, melanin content was measured by Fontana & Masson staining. (**B**) Effect of DGD on α-MSH-induced melanogenice protein expression in HEMn cells. The cells were treated with DGD at the indicated concentrations (0, 0.1 0.5, 1 μM) for 1 h before being exposed to 100 nM α-MSH and harvested 24 h later. Cell lysates were then determined to western blot. The data are representative of three independent experiments that gave similar results.

Several experiments have shown that DGD could be a good agent of depigmentation in melan-a cells. Thus, anti-melanogenic effect of DGC, DGD and IAA were compared in α-MSH-treated HEMn cells using Fontana-Masson staining. Arbutin, a widely known whitening agent was used as positive control (Fig. 6A, i). Treatment with DGD significantly reduced the intercellular melanin production of HEMn cells in a dose-dependent manner (Fig. 6A, e-f) than arbutin at 200 μM. Also we examined whether DGD treatment influence melanogenic protein expression in α-MSH-induced HEMn cells. Consistently with above results in melan-a cells, DGD suppressed tyrosinase and TRP-1 expression in a dose-dependent manner, but not TRP-2 expression (Fig. 6B).

## Discussion

Licorice plant is a well-known medical herb traditionally used as an oriental medicine to cure inflammation disease or skin disease including pigmentation [16-18]. However, anti-melanogenic effect of licorice flavones such as DGC, DGD and IAA were not clearly known. In the present study, we found that DGC, DGD and IAA inhibited melanogenesis and in vitro and intracellular tyrosinase activity in melan-a cells (Fig. 4). DGD showed the most significant inhibitory effect, followed by DGC and IAA. Therefore, we focused on anti-melanogenic effect of DGD and molecular mechanism. Previous reports have demonstrated the role of melanogenic enzyme including tyrosinase and TRPs [26-28]. Therefore, inhibition of melanogenic enzyme expression has been considered as one of effective strategy for suppression of melanin production. DGD inhibited the expression of tyrosinase and TRP-1, but not TPR-2 in melan-a cells (Fig. 5). Consistently, DGD treatment attenuated α-MSH-induced melanogenesis by suppressing tyrosinase and TRP-1 expression in HEMn cells (Fig. 6).

MITF is a member of the basic helix-loop-helix leucine-zipper families of transcription factors and these levels has been regarded that it is most important factor on cell development, survival and proliferation in melanocyte [29]. Especially, it has been demonstrated that it was regulator of melanogenesis via driving melanocyte-specific gene expression such as tyrosinase, TRP-1 and TRP-2; therefore, the inhibition of MITF lead to the suppression of melanin synthesis through reduced tyrosinase expression [29]. DGD inhibited MITF protein levels in melan-a and HEMn cells but not suppressed phosphorylation of CREB levels (Figs. 5 and 6). Consequently, these results suggest that MITF protein levels might be reduced by DGD through MITF degradation systems.

According to previous studies, MITF expression would be modulated by cAMP level, which is induced by α-MSH, and evaluation of cAMP levels result in activation of MAPK pathway [30, 31]. Several evidence suggest that phosphorylation of MITF at Ser73 and Ser409 is responsible for MITF ubiquitination and proteosome-mediated degradation by MAPK ERK2 and p90 Rsk and inhibition of the ERK2 signal pathway by PD98059, specific ERK inhibitor, lead to increasing of melanin production [32, 33]. For these reason, it would be inferred from MAPK activation can inhibit tyrosinase expression via induced MITF degradation through phosphorylation at Ser73 and Ser409. Also, GSK3β regulate MITF DNA-binding via phosphorylation on Ser298 and it is inhibited by AKT signal pathway which is downstream from PI3K [10]. Inhibition of the PI3K pathway induced melanogenesis in B16 melanoma cell and down-regulation of AKT signaling also lead to melanin synthesis in human G361 melanoma cells [12]. Plus, the specific inhibition of the AKT/PKB pathway by LY294002 stimulates melanin synthesis in B16 melanoma cells [11]. Accordingly, inactivated GSK3β through evaluated phosphorylation of PI3K/Akt leads to the suppression of melanin synthesis. As shown in Figs. 5C and 5D, DGD induced phosphorylation of ERK and PI3K/Akt in a dose-dependent manner. DGD also induced phosphorylation of MITF at Ser73 in melan-a cells. Overall these result indicated that DGD is inducer of phosphorylation of ERK and Akt resulting MITF and tyrosinase downregulation.

In summary, DGD, a novel licorice compound, exhibited the most remarkable inhibitory effect on melanogenesis in murine and human melanocytes. DGD suppressed tyrosinase activity and expression and the subsequent MITF downregulation by activating ERK and Akt signal pathway. Consequently, these results suggest that DGD could be a novel whitening agent.
